# Supplementary figures and images for: Dynamic proteomic changes in soft wheat seeds during accelerated ageing
Source: PeerJ. 2018 Nov 2;6:e5874. doi: 10.7717/peerj.5874 (PMC6216954; doi:10.7717/peerj.5874)

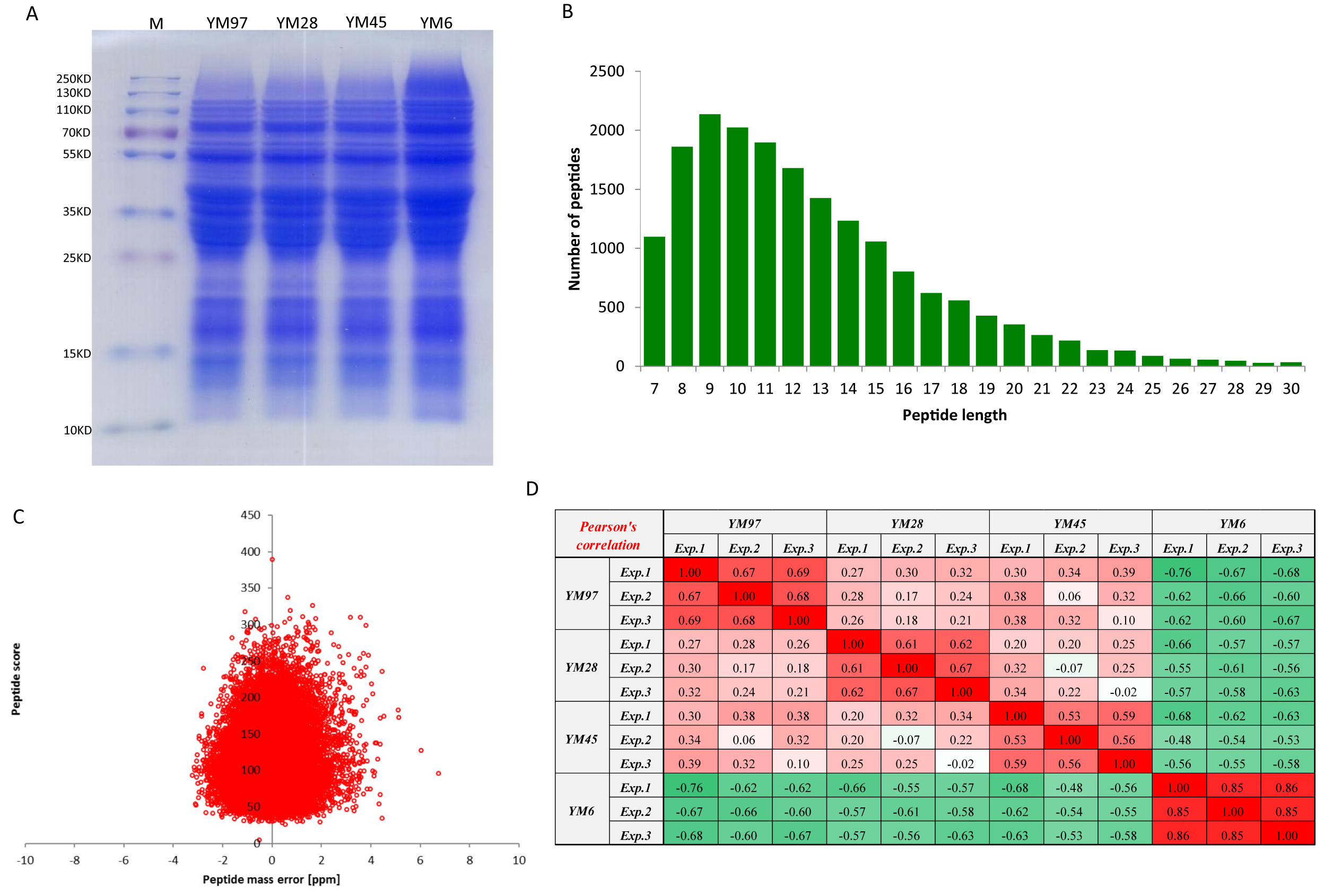

Supplement: Supplemental Information 1 — Figure S1. SDS-PAGE analysis of seed embryos proteins during different storage times (A); Peptide length distribution (B); Mass error distribution of all identified peptides (C); Reproducibility analysis of three repeated trials by Pearson correlation coefficient (D). Table S1. Identification and annotation of proteins during accelerated ageing. [file peerj-06-5874-s001.zip › Supplementary Files/Supplementary Figure 1.tif]
